# Cocaine and Cardiotoxicity: A Literature Review

**DOI:** 10.7759/cureus.14594

**Published:** 2021-04-20

**Authors:** Joseph V Pergolizzi, Peter Magnusson, Jo Ann K LeQuang, Frank Breve, Giustino Varrassi

**Affiliations:** 1 Clinical Research, NEMA Research, Inc., Naples, USA; 2 Cardiology, Centre for Research and Development, Region Gävleborg/Uppsala University, Gävle, SWE; 3 Medicine, Karolinska Institutet, Stockholm, SWE; 4 Scientific Publications, NEMA Research, Inc., Naples, USA; 5 Pharmacy, Temple University, Philadelphia, USA; 6 Research, Paolo Procacci Foundation, Rome, ITA

**Keywords:** cocaine, cocaine-associated angina, cocaine-associated heart failure, myocardial infarction, beta-blockers

## Abstract

Long-term cocaine use, as well as acute cocaine use, is associated with adverse cardiovascular consequences, including arrhythmias, angina, myocardial infarction, heart failure, and other conditions. Over the long term, cocaine can result in structural changes to the heart such as increased left-ventricular mass and decreased left-ventricular end-diastolic volume. Patients arriving with cocaine-associated cardiovascular complaints may not be forthcoming about their cocaine or polysubstance abuse or may be unresponsive. The role of beta-blockers, a first-line treatment for many forms of heart disease, is controversial in this population. Cocaine is a powerful sympathomimetic agent, and it was thought that beta-blockade would result in unopposed alpha-adrenergic stimulation and adverse consequences. A number of small, single-center, retrospective and observational studies suggest that beta-blockers may be safe, effective, and beneficial in this population. Further study is needed to clarify the role of beta-blockers in this population.

## Introduction and background

Cocaine, one of the most frequently consumed recreational drugs, can cause irreversible structural damage to the heart, accelerate cardiovascular disease processes, and trigger arrhythmias and other cardiovascular conditions [1]. In fact, cocaine is the main cause for drug-related visits to the emergency room, and most of these are for cardiovascular problems [2]. Cocaine-associated cardiovascular complications may be further exacerbated by concomitant use of other drugs with cocaine and long-term cocaine use [3]. In a survey on 94 long-term cocaine users (mean regular cocaine use 13.9±9 years), cardiovascular magnetic resonance imaging found that 71% had some form of cardiovascular disease [4].

Cocaine can be smoked (crack cocaine), injected intravenously, insufflated or “snorted,” as well as taken orally or rectally. Since both smoking and intravenous administration have similar pharmacological profiles, smoking a cheap form of “crack” cocaine is particularly popular (Table 1). Snorting cocaine reduces the drug’s bioavailability by more than half because it causes vasoconstriction of the nasal mucus membranes, but snorting is preferred by some users because of the extremely rapid onset of action [1,5].

**Table 1:** Route of administration and key pharmacological data. Source: References [5,6].

Route of Administration	Time to Peak Serum Concentration	Duration of Action
Smoking	1-5 minutes	5-60 minutes
Intravenous injection (cubital fossa)	1-5 minutes	5-60 minutes
Insufflation (snorting)	1 minute	45 minutes
Oral ingestion	45-90 minutes	180 minutes

Cocaine uptake and metabolism occur quickly, producing two major hydrophilic metabolites, benzoylecgonine and ecgonine methyl ester, and a minor metabolite, norcocaine [7]. Cocaine is detectable in oral fluids for about 24 hours, in urine for about four days, and longer in blood, and up to 90 days in hair, although there is interindividual variability [8]. Tolerance, the expected result of long-term cocaine use, may also develop acutely and even to the point that a person under the influence of cocaine feels its effects waning and ingests more while serum concentrations are still high, increasing the risk of potentially life-threatening toxicity [9]. There is such variability in how patients respond to and metabolize cocaine that it is difficult to state what constitutes a lethal dose [9].

A demographic snapshot of the “typical cocaine user” is a male between the age of 35 years and 44 years [10]. Cocaine use occurs among all socioeconomic levels, including high-income individuals, and polysubstance abuse in this population is a common practice [11]. The diversity among cocaine users means they have different underlying risk factors for cardiovascular disease, and although many people actively using cocaine are young and appear healthy, some may be predisposed to certain cardiac conditions that are exacerbated by cocaine [6]. The cocaine-associated myocardial infarction retrospective study evaluated 130 patients with 136 cocaine-associated myocardial infarction events and found the most patients were young men (mean age 38 years), nonwhite (70%), smoker (91%), with a history of having used cocaine in the 24 hours before the event (88%) [12]. In that study, 43% of patients experienced arrhythmias and 7% heart failure [12].

## Review

Cocaine is a powerful sympathomimetic agent because it blocks the reuptake of norepinephrine and dopamine at the pre-synaptic adrenergic terminals, allowing catecholamines to accumulate at the post-synaptic receptor [13]. Thus, cocaine increases both sympathetic output and catecholamine production, which has a dual effect. On the one hand, it increases cardiac rate, blood pressure, and cardiac contractility all of which increase myocardial oxygen demand, and on the other hand, it causes vasoconstriction, platelet adherence, thrombus formation, and coronary spasm, all of which simultaneously decreases oxygen supply [14]. This myocardial oxygen mismatch can lead to ischemia and infarction. Cocaine can also increase blood pressure and heart rate in a dose-dependent fashion [15]. Alcohol and tobacco use are common among cocaine users; alcohol amplifies these chronotropic effects [16], and nicotine can exacerbate pre-existing coronary artery disease and ischemia [17].

Cocaine stimulates the release of the potent vasoconstrictor endothelin-1 along with the alpha-adrenergic receptors of the smooth muscle cells of the coronary arteries, but endothelin-1 inhibits the synthase of the vasodilator, nitric oxide [14]. Endothelin-1 and nitric oxide are natural counterparts for healthy vascular function and the inducing of endothelin-1 and inhibition of nitric oxide creates an imbalance that promotes vasoconstriction, elevated blood pressure, and potential vascular remodeling and dysfunction [18]. Cocaine also reduces sodium transport, which is responsible for its effects as a local anesthetic [14]. Sodium channel blockade can prolong the QT and QRS intervals, trigger arrhythmias, and cause systolic dysfunction.

Structural changes in the heart may be the result of long-term cocaine use; this is the physiologic reaction to cocaine-associated hypertension, increasing vascular resistance, and causing the myocardium, particularly the left ventricle, to increase its mass as a compensatory mechanism. In a meta-analysis, chronic cocaine use could be associated with increased heart weight [19]. In a study on 20 chronic cocaine users and 20 healthy controls, long-time cocaine users had significantly greater left-ventricular mass than controls (18% greater, 124±25 vs 105±16 g, p=0.01), independent of body surface area [20]. This increased left-ventricular mass was statistically associated with the duration and frequency of cocaine use [20]. Cocaine may be associated with decreased left-ventricular end-diastolic volume [19]. This may be due to the fact that cocaine causes small-vessel coronary dysfunction and/or direct myocardial damage [2]. Table 2 shows other cardiac conditions that may be associated with or exacerbated by cocaine use.

**Table 2:** Cardiovascular conditions that may be associated with cocaine use. The most frequently reported cocaine-associated symptom is chest pain (angina), although conditions such as cocaine-induced arrhythmias are likely under-reported. In the United States in 2016, it was estimated that 0.8% of the population over 18 was currently using cocaine; the lifetime prevalence of cocaine use was 3.3% for Caucasians and 5.0% for Blacks. Source: References [1, 2, 4, 6, 12, 14, 19, 21-36]. ECG: electrocardiogram

Symptom	Description/Mechanism	Comments
Aortic Dissection	Cocaine adversely affects aortic connective tissue and cocaine-induced hypertension may increase shear stress that penetrates the intimal vessel layer, allowing blood to separate intimal and medial layers.	Cocaine-associated aortic dissection is rare ~ 1% of all aortic dissection.
Arrhythmias	Cocaine blocks potassium channels increases L-type calcium channel currents and inhibits the inflow of sodium during depolarization. Cocaine dissociates gradually from sodium channels and excitability is not restored quickly which can predispose to conduction problems and re-entry circuits. Catecholamine toxicity may cause micro focal damage to the myocardium and necrotic myocytes may appear and fibrosis, impeding normal conduction.	Cocaine-induced arrhythmias are likely under-reported and ECG abnormalities among cocaine users are so variable that they may be considered idiosyncratic. Some ECG changes resemble those caused by Brugada syndrome (right bundle-branch block with ST-segment elevation in V1, V2, and V3). Types of arrhythmias associated with cocaine: supraventricular tachycardia, sinus bradycardia, accelerated idioventricular rhythms, bundle-branch block, complete heart block, monomorphic ventricular tachycardia, torsades-de-pointes, and ventricular fibrillation.
Cardiomyopathy and Heart Failure	Chronic cocaine use is associated with anatomical and functional changes to the heart that resemble diastolic heart failure. Cocaine-produced reactive oxygen species overwhelms the myocardial antioxidant defenses.	Toxic cardiomyopathies, such as those related to cocaine use, are only now starting to be elucidated In a study of 430 consecutive dilated cardiomyopathy patients, 2.3% (n=10) were considered cocaine-related. Long-term cocaine users are at elevated risk for heart failure.
Chest pain	Cocaine use may induce immediate chest pains, but chest pain (angina) may not be indicative of myocardial infarction although a 12-hour observation period is recommended.	The most common cocaine-related cardiovascular symptom; 57% of cocaine users with chest pains are admitted to hospital.
Coronary Artery Disease and Aneurysm	Cocaine disrupts endothelial activity.	Overall, in patients undergoing angiography, coronary artery aneurysm occurs at the rate of 0.2% to 5.3%, but coronary artery aneurysms are significantly more prevalent among cocaine users (30.4% vs 7.6%, p<0.001).
Endocarditis	Increased heart rate and blood pressure may lead to valvular and vascular injury that can predispose to bacterial invasion	Cocaine appears to be a risk factor for infective endocarditis.
Hypertension	Adrenergic stimulation increases the norepinephrine effect at post-synaptic receptor sites which can trigger hypertension and tachycardia, increase myocardial oxygen demand, and reduce oxygen supply via vasoconstriction.	Acute hypertensive crises possible in this population.
Myocardial Infarction	Vasoconstriction and increased myocardial oxygen demand accelerate atherosclerosis and promote thrombus formation.	Cocaine users are at a 7-fold risk for myocardial infarction compared to non-users The CHOCHPA study found 6% of emergency room patients treated for chest pain had a cocaine-induced myocardial infarction.
Valvular Disease	Vasoconstriction, endothelial dysfunction, accelerated atherosclerosis can cause valvular damage over time.	Chronic cocaine use may lead to damaged vessels and valves.

These cocaine-associated symptoms can be exacerbated by behavioral, lifestyle, and environmental factors typical of the drug users. The prevalence of alcohol and tobacco use is high among cocaine users and may worsen vascular toxic effects. Cocaine users may take other illicit drugs and the complications of polysubstance abuse are only beginning to be elucidated. Cocaine, like other drugs, maybe mixed with potentially toxic adulterants. For those whose lives are taken over by substance use disorder, compounding their problems are environmental risk factors such as poor nutrition, homelessness, legal problems, and exposure to potentially violent situations.

Cocaine may cause cardiovascular conditions, exacerbate pre-existing cardiovascular conditions, and may adversely influence patient compliance, therapy, and cardiovascular management strategies [35]. In this connection, it may be important to query patients about possible cocaine use in the context of cardiovascular conditions. While the demographic portrait of the typical cocaine user is a young, apparently otherwise healthy, man, there is also a geriatric subpopulation of patients with cocaine-induced cardiovascular conditions. The great heyday of cocaine abuse in the United States occurred in the 1980s and whether or not these people have continued to use cocaine or other substances, they may be suffering cocaine-induced cardiac conditions [37]. Thus, as the “cocaine generation” of the 1980s ages, there is a public health need to better recognize and treat those with cocaine-impaired cardiovascular systems in addition to treating younger people who have cocaine use disorder. While some cocaine-associated symptoms, such as chest pain and myocardial infarction, typically occur immediately, other conditions such as atherosclerosis develop over long periods of chronic drug use with few, if any, early symptoms [35]. For older patients with a history of a long-term cocaine use disorder, it is clinically useful to consider possible cardiovascular disorders.

Most clinical encounters with cocaine-associated cardiovascular conditions occur in emergency medicine with acute conditions (chest pain, myocardial infarction, arrhythmias) in young patients with few apparent risk factors for heart disease. This paradoxical patient with a cardiovascular emergency may be experiencing cocaine-associated complications. Their care can be complex, because cocaine-intoxicated patients may arrive unresponsive or minimally responsive, and even if responsive, may not be forthcoming about cocaine or other illicit drug use. They may admit to having taken drugs but not know exactly what they had consumed, they may have taken multiple substances (polysubstance abuse), or they may have taken illicit drugs with unknown and potentially dangerous adulterants [3]. Clinicians may like to consider asking young male patients in apparent good health seeking emergency care for chest pain about possible cocaine use. When evaluating a patient with suspected myocardial infarction and possible cocaine use, the cardiac troponin values are sensitive and specific markers for cocaine-induced myocardial infarction [10,38]. However, even without positive cardiac troponin values, it is prudent to allow a nine-to 12-hour observation period in a chest pain observation unit before discharging such patients [10]. Only a small number of cocaine users with chest pains have a myocardial infarction, but it should be ruled out even in young patients. Cocaine users presenting with chest pain, unstable angina, or myocardial infarction should be treated according to protocols for acute coronary syndrome [39,40].

Cocaine is associated with arrhythmias which may cause moderate to severe symptoms, but there are no specific treatments for cocaine-induced arrhythmias [25]. Lidocaine should be administered with caution, if at all, because it may lower the patient’s seizure threshold [25]. Calcium-channel blockers may be useful [25]. However, in those with cocaine use, intravenous benzodiazepines may be appropriate to help relieve chest pain and improve cardiac hemodynamics and improve potential neuropsychiatric symptoms. Nitroglycerin may be effective at relieving cocaine-induced chest pain by reversing cocaine-related vasoconstriction [41,42]. Further study is warranted to expand the arsenal of treatments for cocaine-induced cardiovascular conditions.

Beta-blockers in this population

The role of beta-blockade in treating the cardiovascular consequences of cocaine use remains controversial. The reservation about beta-blockers centers on unopposed alpha-adrenergic stimulation, which has not been frequently observed [43]. Since beta-blockers are a mainstay treatment for heart failure and other cardiovascular conditions which are prevalent among the population of active cocaine users, clinicians may be unsure of their role specifically for cocaine users. Furthermore, clinicians who conclude that beta-blockers are best avoided in cocaine users may need to develop tools to screen patients for cocaine use rather than prescribing these drugs under the assumption that the patient is not simultaneously taking cocaine.

There is some weak evidence in support of the use of beta-blockers among cocaine users with heart disease. In a systematic review (n = 1994 from 12 studies), it was found that beta-blockers conferred either a beneficial or a neutral effect on primary outcomes in heart failure in patients actively using cocaine [44]. It should be noted that there are no large-scale studies on beta-blockade in active cocaine users and the information from this meta-analysis comes mainly from small, single-center, retrospective studies [44]. Furthermore, this study evaluated patients with heart failure, which is not the only clinical cardiovascular manifestation of long-term cocaine use.

Another consideration is whether or not some beta-blockers may be better than others for cocaine users with cardiovascular disease. There are several types of beta-blockers, and no studies have specifically identified if certain beta-blockers are more appropriate than others for this population. Nonselective beta-blockers block alpha as well as beta receptors. In an observational study of 2578 patients admitted to a hospital for acute heart failure, 20% (n = 503) had cocaine-associated heart failure [45]. The primary endpoint of this study was a major adverse cardiac event (MACE), including a 30-day heart failure hospital re-admission. Of the cocaine-associated heart failure patients, 404 were taking carvedilol and 99 were not taking any beta-blocker. At baseline, carvedilol patients had lower ejection fraction scores, lower heart rates, lower N-terminal-pro hormone B-type natriuretic peptide (NT-proBNP) concentrations, and more coronary artery disease. Over 19 months of follow-up, 169 patients in the study reported a MACE. Using multivariate analysis, the independent predictors of MACE among those actively using cocaine were history of coronary artery disease, lower ejection fraction, elevated pulmonary artery systolic pressure, higher NT-proBNP levels, less education, current unemployment, and less use of standard heart failure medical therapy. It should be noted that the rates for MACE were similar in patients using or not using carvedilol. Among those with an ejection fraction <40%, there was a significantly lower rate of MACE in those taking carvedilol than not (34% vs 58%, p = 0.02), but in patients with an ejection fraction >40%, MACE rates were similar among those taking and those not taking carvedilol. Thus, carvedilol may be effective for active cocaine users with cocaine-associated heart failure and ejection fractions <40% [45]. The particular interest in this study was that the type of cocaine and frequency of cocaine use did not seem to influence the results [45]. Of course, this study is limited in that it was an observational, single-center study and only carvedilol was used due to institutional practices.

There are no guidelines about the use of beta-blockers in cardiac patients actively using cocaine. The 2014 Guidelines from the American Heart Association and the American College of Cardiology advise against the use of beta-blockers in patients with acute cocaine toxicity because they could potentiate coronary spasm [46]. However, this guidance allows that beta-blockers may be used in patients with non-ST elevation acute coronary syndrome with recent cocaine use [46]. Havakuk and colleagues recommend considering beta-blockers for heart failure, arrhythmias, and other cardiovascular conditions [2].

The role of beta-blockers specifically for the acute coronary syndrome (ACS) remains controversial. The argument is made that since beta-blockers also block alpha receptors, they may result in a decreased supply of oxygen-rich blood to the myocardium, exacerbating ACS [47]. However, this report is limited in that it drew on evidence from only two small studies, one from 1990 and the other from 1993, n = 30 and 15, respectively. In a 2010 study on 90 acute coronary syndrome patients with a positive urine assay for cocaine were administered standard ACS therapy and administered either labetalol (n = 60) or diltiazem (n = 30) [48]. Both groups experienced a significant and similar decrease in blood pressure after 48 hours. After 48 hours, the labetalol patients had better hemodynamic parameters and no adverse events, allowing investigators to conclude labetalol was effective in ACS patients with cocaine use disorder [48].

A meta-analysis published in 2019 of five studies (n = 1447) found no differences between patients with cocaine-associated chest pain treated with beta-blockers and those not treated with beta-blockers in terms of the risk for myocardial infarction (relative risk {RR} 1.08, 95% confidence interval {CI} 0.61-1.91) or all-cause mortality (RR 0.75, 95% CI, 0.46-1.24) [49]. Thus, beta-blockers were not associated with adverse outcomes in cocaine-associated chest pain.

The crucial question about beta-blockade for patients actively using cocaine is an important one because beta-blockers are known to confer morbidity and mortality benefits to people with cardiovascular disease. Therefore, withholding these drugs to patients with cocaine-associated heart disease is an important decision and the evidence about their risks and benefits is not robust. Further study is needed to answer more questions: are beta-blockers safe and effective for people actively using cocaine? Can we quantify their risks and their morbidity and mortality benefits? Are particular beta-blockers or treatment regimens safer and more effective than others for this population?

## Conclusions

Cocaine use is prevalent and associated with cardiovascular adverse effects. Cocaine may cause acute complications, including arrhythmias, chest pain, and myocardial infarction, but long-term cocaine use may also be associated with valvular disease, structural heart disease, and ischemic heart disease. It is important to consider cocaine as a potential complicating factor in heart disease. While the typical cocaine user is a young male, the population of current and former cocaine users is quite heterogeneous.
